# Protective Effects of *Lycium ruthenicum* Murray against Acute Alcoholic Liver Disease in Mice via the Nrf2/HO-1/NF-κB Signaling Pathway

**DOI:** 10.3390/ph17040497

**Published:** 2024-04-13

**Authors:** Niantong Xia, Zimian Ding, Mingran Dong, Shuyang Li, Jia Liu, Hongwei Xue, Zhigang Wang, Juan Lu, Xi Chen

**Affiliations:** 1 State Key Laboratory for Quality Ensurance and Sustainable Use of Dao-di Herbs, Institute of Medicinal Plant Development, Chinese Academy of Medical Sciences, Peking Union Medical College, Beijing 100193, China; 18263825319@163.com (N.X.); zmding@implad.ac.cn (Z.D.); dongmingran1998@163.com (M.D.); 15563999708@163.com (S.L.); lj200929@126.com (J.L.); 15297318065@163.com (H.X.); 2 Department of Pharmaceutical Analysis, Heilongjiang University of Chinese Medicine, Harbin 150040, China; wangzhigang0513@vip.163.com

**Keywords:** alcoholic liver disease, *Lycium ruthenicum* Murray, anthocyanins, oxidative stress, inflammatory response, Nrf2/HO-1/NF-κB pathway

## Abstract

Acute alcoholic liver disease (ALD) resulting from short-term heavy alcohol consumption has become a global health concern. Moreover, anthocyanins have attracted much attention for their ability to prevent oxidation and inflammation. The present work evaluates the protective effects of *Lycium ruthenicum* Murray (LRM) against ALD and explores the possible underlying mechanism involved. The total anthocyanin content in LRM was 43.64 ± 9.28 Pt g/100 g dry weight. Mice were orally administered 50, 125, or 375 mg LRM/kg body weight (BW) for 21 days. On days 18–21, mice were orally administered 15 mL of ethanol/kg BW. Markers of liver damage, oxidative stress, and inflammation were examined. Furthermore, the modulatory effect of LRM on Nrf2/HO-1/NF-κB pathway molecules was evaluated through quantitative reverse transcription polymerase chain reaction (RT‒qPCR) and immunohistochemistry analyses. The difference between the groups indicated that LRM improved liver histopathology and the liver index, decreased aspartate transaminase, alanine transaminase, malondialdehyde, reactive oxygen species, IL-6, TNF-α, and IL-1β expression, but elevated superoxide dismutase, catalase, and glutathione-s-transferase levels. Moreover, LRM upregulated *Nrf2* and *Ho-1* but downregulated *Nf-κb* and *Tnf-α* genes at the transcript level. In summary, LRM alleviated ethanol-induced ALD in mice by reducing oxidative damage and associated inflammatory responses. LRM protects against ALD by reducing damage factors and enhancing defense factors, especially via the Nrf2/HO-1/NF-κB pathway. Thus, LRM has application potential in ALD prophylaxis and treatment.

## 1. Introduction

Short-term heavy drinking can cause various alcohol-related diseases and acute alcoholism [1,2]. Alcoholic liver disease (ALD) and acute alcoholism are major public health issues worldwide, as are cancer and cardiovascular diseases. The accumulation of large amounts of alcohol, a true liver toxin, and its intermediate metabolites in the liver can cause chronic liver damage [3]. ALD results from short-term excessive alcohol consumption, which may cause steatosis, alcoholic hepatitis, cirrhosis in the early stages, and liver cancer in severe cases. The liver represents a major alcohol metabolic site within the human body [4]. Ethanol metabolism-related oxidative stress, glutathione consumption, cell damage, autophagy, inflammation, regeneration, bacterial translocation, and gut-liver axis dysbiosis exert critical effects on ALD’s pathogenic mechanism [5,6,7,8]. The mechanism of ALD development mainly involves oxidative stress and inflammation. Two crucial regulatory systems, the nuclear factor erythroid 2-related factor 2 (Nrf2)/heme oxygenase-1 (HO-1) signaling pathway and the nuclear factor κB (NF-κB) system, interact to regulate intracellular redox balance and inflammatory response [9,10,11].

LRM belongs to the *Solanaceae* family and contains anthocyanins, which are water-soluble polyphenols that possess high antioxidant and free radical scavenging activities [12,13,14]. Thus, it helps the body protect against oxidative stress and reduce free radical damage [15,16,17]. Dong Mingran et al. [18] discovered that LRM can activate the antioxidant pathway SIRT1/Nrf2/Keap1, thereby reducing the reproductive toxicity caused by exposure to the heavy metal Cd by easing oxidative stress in the testes. LRM likely modulates inflammatory factors, such as tumor necrosis factor-α (TNF-α) and interleukin-6 (IL-6) [19,20,21], and inhibits inflammatory pathways with its bioactive components. Yujia Peng et al. [22] discovered that LRM enhanced the integrity of the gut barrier, suppressed the signaling pathway of Toll-like receptor 4 (TLR4), and ameliorated neuroinflammation. Hu Ge et al. [23] revealed that LRM modulated the TLR4/p38 MAPK signaling cascade to alleviate inflammatory stress and mitigate liver damage induced by alcohol in rats. Furthermore, Baoming Tian et al. [24] demonstrated that LRM enhances oxidative stress by activating the Nrf2/HO-1/NQO1 pathway and reduces liver inflammation by downregulating TLR4/NF-κB/JNK in liver tissue. As a result, the modulation of inflammation and oxidative stress by LRM via the Nrf2/HO-1/NF-κB pathway may be a promising therapeutic target for ALD and offer novel insights into the mechanisms through which LRM prevents ALD [24].

This work analyzed the possible protective effect of LRM on ALD and evaluated its ability to reduce liver injury, oxidative stress, and inflammation. Furthermore, we explored the possible mechanism by which LRM confers protection against ALD.

## 2. Results

### 2.1. LRM Chemical Components

The total anthocyanin content in LRM was 43.64 ± 9.28 Pt g/100 g dry weight, indicating that LRM is an important anthocyanin source with potential clinical application.

### 2.2. Effect of LRM on the Mouse Liver

**Figure 1:**
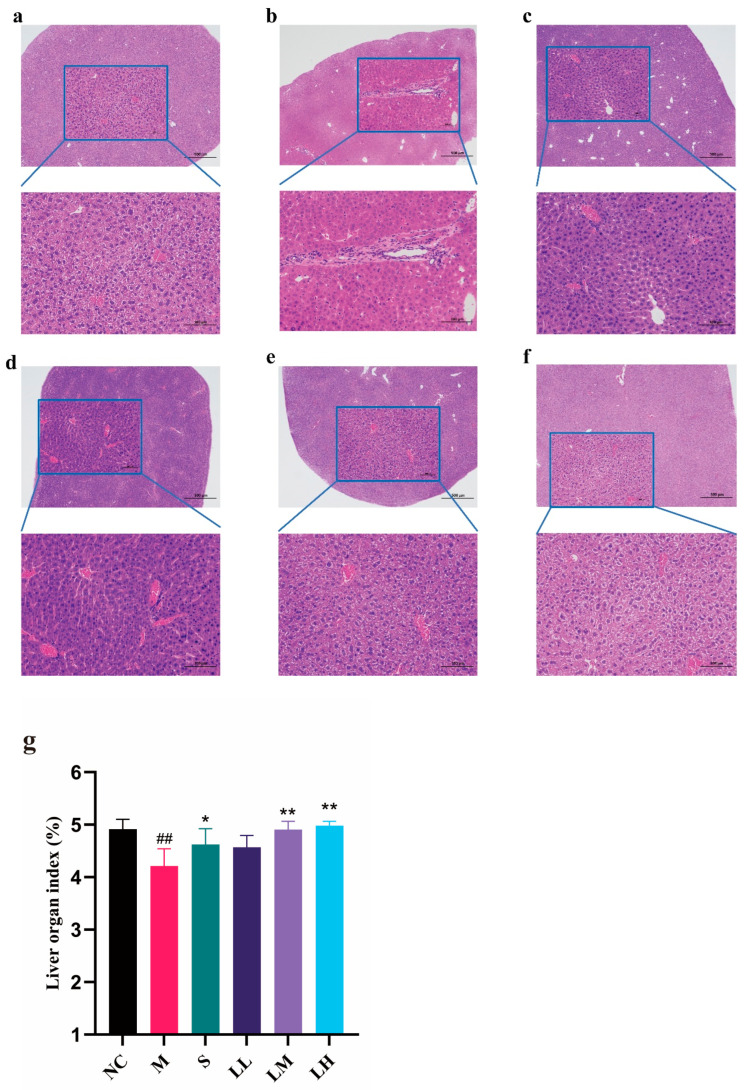
Histopathological sections from each group. (means ± SD, *n*= 6). (**a**) NC. Scale bar: 100 μm; (**b**) M. Scale bar: 100 μm; (**c**) S. Scale bar: 100 μm; (**d**) LL. Scale bar: 100 μm; (**e**) LM. Scale bar: 100 μm; (**f**) LH. Scale bar: 100 μm; (**g**) liver organ indices. ## *p* < 0.01 vs. the normal control group; * *p* < 0.05, ** *p* < 0.01 vs. the model group.

As shown in Figure 1a, the NC group had a normal liver structure with an intact cytoplasm, distinct nuclei, and clear veins. In the M group (Figure 1b), inflammatory infiltrates, hepatocyte degeneration, hepatic sinusoid dilation, and liver tissue congestion were observed. By contrast, liver tissue from mice in the S group (Figure 1c) and LRM group (Figure 1d–f) presented reduced levels of inflammatory infiltrates, hepatocyte degeneration, and hemorrhage compared to untreated groups. LRM dose-dependently exerted hepatoprotective effects. Moreover, hepatoprotection in the LM and LH groups was similar to that in the S group. The M group’s liver weight and body mass index decreased (*p* < 0.01) relative to those of the NC group. Interestingly, the LRM-treated groups had higher liver weights and body mass indices than the M group (*p* < 0.01).

### 2.3. Role of LRM in Serum Biochemical Indicators of Alcohol-Mediated Liver Injury in Mice

**Figure 2:**
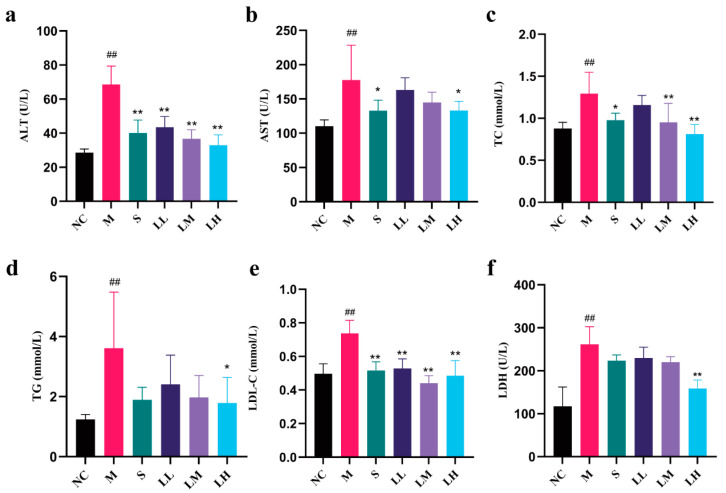
Alterations in the serum ALT, AST, TC, TG, LDL-c, and LDH contents among C57BL/6J mice after administration of 15 mL alcohol/kg body weight. Red star Erguotou liquor at 56° (means ± SD, *n* = 6) (**a**) ALT; (**b**) AST; (**c**) TC; (**d**) TG; (**e**) LDL-C; (**f**) LDH. ## *p* < 0.01 vs. the normal control group; * *p* < 0.05, ** *p* < 0.01 vs. the model group.

The role of LRM in alcohol-mediated liver injury in mice is shown in Figure 2. Compared to those in the NC group, the M group had significantly higher serum levels of ALT, AST, TC, TG, LDH, and LDL-C (*p* < 0.01). The S group demonstrated obvious reductions in the serum ALT, TC, TG, and LDL-C levels (*p* < 0.01) and nonsignificant improvements in the serum AST and LDH levels compared to those in the M group. Moreover, compared to those in the NC group, the serum levels of ALT, LDL-C, and TC in the LRM-treated group were significantly lower (*p* < 0.01). Moreover, TG levels were significantly lower in the LH group than in the M group (*p* < 0.05).

### 2.4. Role of LRM in Oxidative Damage in the Liver

**Figure 3:**
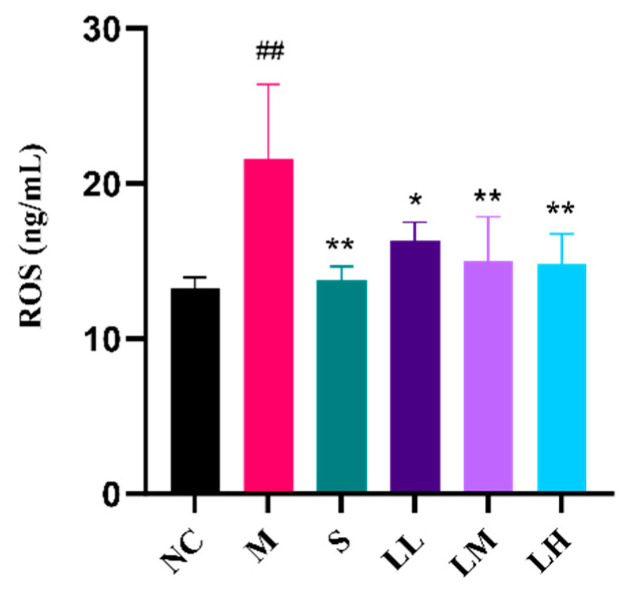
Changes in serum ROS concentration in C57BL/6J mice after administering 15 mL of ethanol/kg body weight. (means ± SD, *n* = 6). ## *p* < 0.01 vs. the normal control group; * *p* < 0.05, ** *p* < 0.01 vs. the model group.

As shown in Figure 3, the M group exhibited markedly increased ROS content following ethanol administration relative to that in the NC group. Following ethanol administration, the mice in the S group showed decreased ROS levels relative to those in the M group. Compared to those in the M group, ROS levels in the LRM-treated group decreased in a dose-dependent manner following ethanol administration. Overall, LRM can promote antioxidant defense, inhibit ROS production in the body, and alleviate alcohol-mediated oxidative stress.

**Figure 4:**
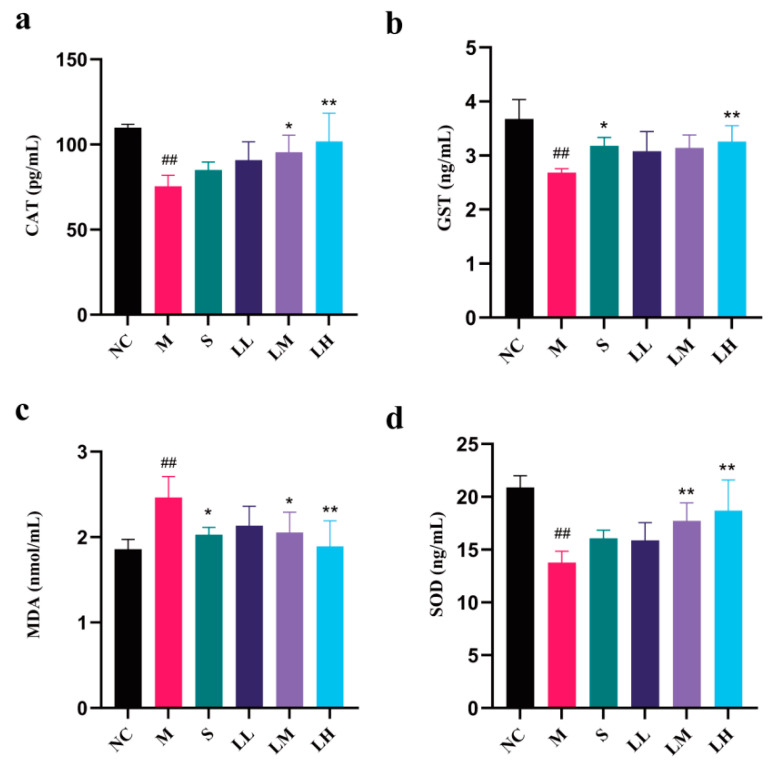
Changes in CAT, GST, MDA, and SOD contents in liver tissue from C57BL/6J mice after administering 15 mL of ethanol/kg body weight. (means ± SD, *n* = 6). (**a**) CAT content; (**b**) GST content; (**c**) MDA content; (**d**) SOD content. ## *p* < 0.01 vs. the normal control group; * *p* < 0.05, ** *p* < 0.01 vs. the model group.

The effects of LRM on SOD, MDA, CAT, and GST contents in liver tissue homogenates from liver injury model mice are presented in Figure 4. SOD, CAT, and GST levels were significantly lower in the M group than in the NC group (*p* < 0.01), but dramatically elevated MDA and ROS levels were detected in the M group (*p* < 0.01). Silibinin and high-dose LRM had regulatory effects on SOD, MDA, CAT, and GST levels in liver tissue (*p* < 0.01). These results demonstrate dose-dependent LRM activity.

### 2.5. Role of LRM in Inflammatory Cytokines and Mediators of Alcohol-Induced Liver Injury in Mice

**Figure 5:**
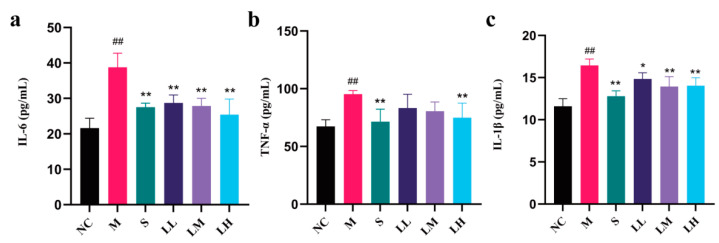
Changes in IL-6, TNF-α, and IL-1β levels in liver tissue from C57BL/6J mice after administering 15 mL of ethanol/kg body weight. (means ± SD, *n* = 6). (**a**) IL-6; (**b**) TNF-α; (**c**) IL-1β levels. ## *p* < 0.01 vs. the normal control group; * *p* < 0.05, ** *p* < 0.01 vs. the model group.

Figure 5 shows LRM’s effects on TNF-α, IL-6, and IL-1β levels in liver tissue. Compared to those in the NC group, markedly elevated TNF-α, IL-6, and IL-1β levels were observed in the M group (*p* < 0.01). Compared to those in the M group, the TNF-α, IL-6, and IL-1β levels in the S group were markedly lower (*p* < 0.01), while those in the LH group were significantly lower (*p* < 0.01), indicating that LRM exhibited dose-dependent activity.

### 2.6. Role of LRM in Nrf2, HO-1, NF-κB, and TNF-α

**Figure 6:**
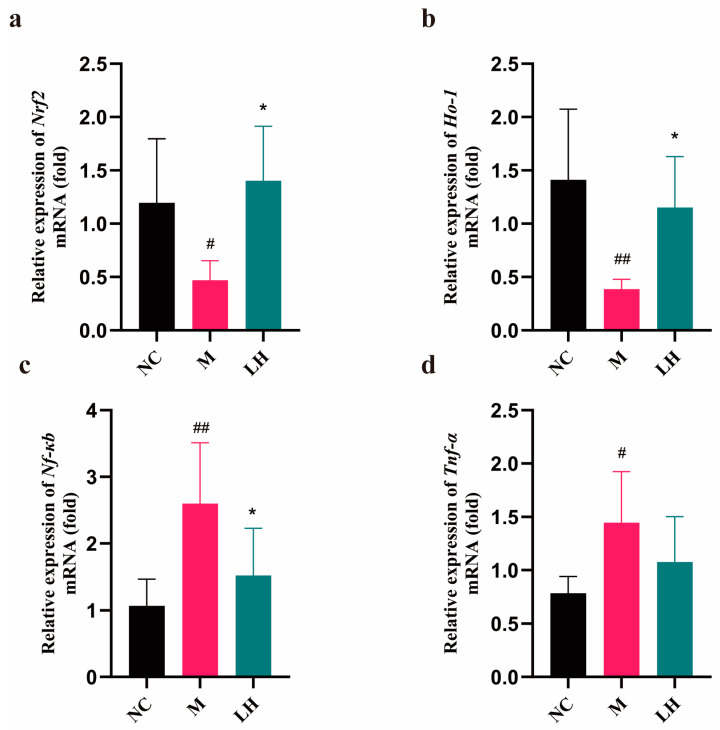
mRNA expression in the normal control group. (means ± SD, *n* = 6). (**a**) *Nrf2*, (**b**) *Ho-1*, (**c**) *Nf-κb*, (**d**) *Tnf-α*. # *p* < 0.05, ## *p* < 0.01 vs. the normal control group; * *p* < 0.05 vs. the model group.

According to Figure 6, relative to those in the NC group, the *Nrf2* and *Ho-1* levels in the livers of mice in the M group were evidently decreased, but the *Nf-κb* and *Tnf-α* expression levels were markedly elevated (*p* < 0.01). Compared to those in the M group, *Nrf2* and *Ho-1* levels in the liver dramatically increased, while *Nf-κb* levels markedly decreased in the LH group (*p* < 0.01). Moreover, *Tnf-α* activity in the LH group decreased relative to that in the M group. The above findings suggest that LRM can reduce the mRNA levels of the studied genes in the livers of mice with alcoholic hepatitis. Specifically, LRM promotes *Nrf2* activation, leading to *Ho-1* upregulation. Thus, LRM enhances cells’ antioxidant capacity. Moreover, activated *Nrf2* plays an anti-inflammatory role by suppressing *Nf-κb* and *Tnf-α*, slowing the inflammatory response.

**Figure 7:**
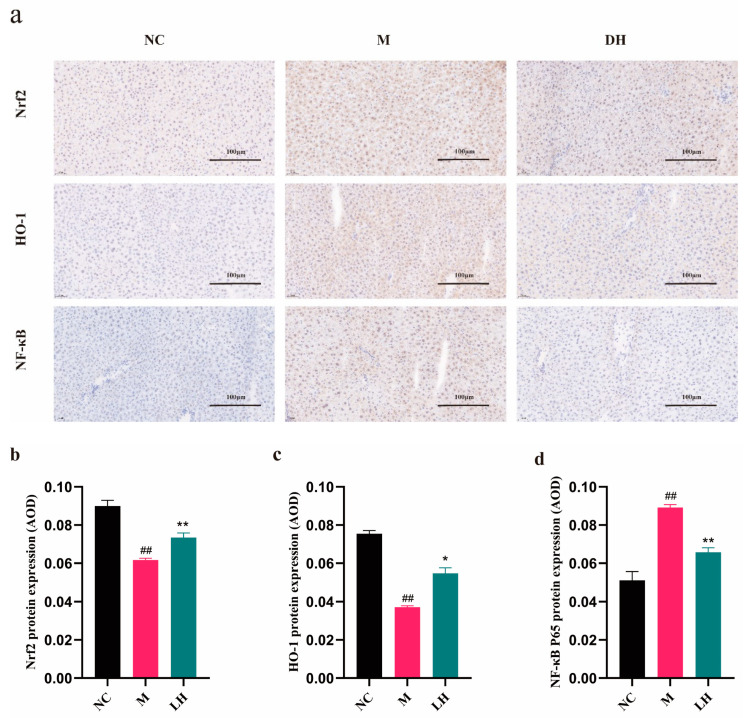
Immunohistochemical staining analysis of Nrf2, HO-1, and NF-κB in each group. (means ± SD, *n* = 6). (**b**) Nrf2, (**c**) HO-1, (**d**) NF-κB. ## *p* < 0.01 vs. the normal control group; * *p* < 0.05, ** *p* < 0.01 vs. the model group.

Positive results are indicated by brown-yellow regions (Figure 7a), suggesting antigen antibodies binding to liver cells. The average density was determined by dividing the integrated optical density value (IOD) by the pixel area (AREA) of each positive image, allowing for the analysis of immunohistochemical findings through the mean density (AOD) for protein quantification [25]. Figure 7b–d displays the results of immunofluorescence staining for Nrf2, HO-1, and NF-κB in hepatic tissue sections. Compared to those in the NC group, NF-κB expression increased, while Nrf2 and HO-1 expression decreased in the M group. By contrast, Nrf2 and HO-1 expression was upregulated (*p* < 0.05, or *p* < 0.01) while NF-κB expression decreased after LRM treatment (*p* < 0.01). These findings confirmed the RT-qPCR results. Additionally, the LH group exhibited elevated relative fluorescence intensities of Nrf2 and HO-1, which are important molecules in the cellular antioxidant defense system (*p* < 0.05). LRM activated the Nrf2/HO-1 pathway while inhibiting the NF-κB pathway, reversing acute alcohol-mediated effects.

## 3. Discussion

At present, traditional Chinese medicine is increasingly used to treat ALD [26,27,28,29]. ALD results from excessive alcohol consumption and greatly threatens the liver [30,31,32]. Chinese medicine, as a comprehensive treatment, plays a crucial role in liver protection [33], particularly via anti-inflammatory and antioxidative effects [34,35,36]. These effects alleviate the burden on the liver, promoting liver cell repair and inhibiting ALD development. An increasing number of studies have reported that ALD development is related to oxidative stress [37] and inflammatory responses [38], which can be alleviated by LRM [39,40]. However, LRM’s efficacy in treating alcohol-induced liver injury remains unclear. Therefore, this study constructed an ALD mouse model to elucidate LRM’s therapeutic effect and identify its possible mechanism of action. Since silibinin has good therapeutic effects on liver injury, it was used as a positive control. Multiple liver protective effects of LRM were observed in a mouse model of ALD, such as reducing oxidative damage and inflammation caused by ALD.

As the main alcohol-metabolizing organ, the liver may suffer varying degrees of alcohol-induced damage in cases of excessive alcohol consumption, especially in the early stages of ALD; however, this damage can be reversed [41,42]. Histopathological examination of liver tissue, especially hematoxylin and eosin staining, revealed severe effects of excessive alcohol consumption. In the M group, inflammatory infiltrates, hepatocellular degeneration, and hyperemia of the hepatic sinuses were observed. However, LRM ameliorated these symptoms and improved the liver index, demonstrating its hepatoprotective effects. Liver enzymes, such as AST and ALT, are sensitive and specific serum biomarkers of early liver injury [43,44]; serum TG content is also an indicator of hepatic steatosis [45]. Excessive alcohol intake hinders fatty acid oxidation and the tricarboxylic acid cycle, affecting fat metabolism. Therefore, TG accumulates within the liver, increasing its blood level [46,47]. The liver is also the main organ of cholesterol synthesis. Liver injury affects its ability to synthesize and metabolize cholesterol, resulting in increased serum TC and LDL-C levels [48]. LDH is a cytoplasmic enzyme involved in lactic acid metabolism that indicates cell damage [49]. In our study, serum AST, ALT, TC, LDL-C, TG, LDH, and other biochemical markers were significantly increased in the M group. However, LRM treatment significantly decreased these markers’ serum levels and restored metabolic activity. LRM reduced serum LDH concentration in a dose-dependent manner, indicating its ability to protect against alcohol-mediated hepatocyte injury and ameliorate steatosis.

In liver cells, alcohol can be initially metabolized into acetaldehyde before being subsequently oxidized into acetate by acetaldehyde dehydrogenase [50,51]. Excessive alcohol consumption causes acetaldehyde and ROS accumulation, resulting in oxidative stress [52,53]. Oxidative stress-induced liver damage, which includes lipid peroxidation, protein oxidation, antioxidant system activity, and mitochondrial dysfunction, ultimately affects liver structure and function by inducing inflammation and apoptosis and exacerbating liver damage [54,55]. Excessive ROS production due to excessive alcohol consumption impairs the levels of antioxidant enzymes in the liver, such as SOD, CAT, and GST, disrupting the removal of free radicals and other oxidative substances [56]. MDA content is a crucial factor indicating the body’s potential antioxidant capacity. It reflects the speed and strength of lipid peroxidation within the body, and can indirectly reveal the extent of tissue damage caused by peroxidation [57]. TNF-α, IL-1β, and IL-6 are common proinflammatory agents used to treat alcohol-mediated liver injury. Alcohol stimulates NF-κB pathway activation within liver macrophages, inducing the expression of these factors [58,59,60]. In the present study, LRM decreased ROS and MDA but increased SOD, CAT, and GST. Thus, LRM alleviates oxidative stress by promoting antioxidant enzyme generation and stabilizing their active centers or reducing their degradation. In our study, LRM reduced TNF-α, IL-1β, and IL-6 concentrations in hepatocytes, confirming its anti-inflammatory effects.

Oxidative stress and inflammation are key factors for the onset and development of alcohol-mediated liver injury [38,61,62]. The NF-κB and Nrf2/HO-1 pathways are important for regulating intracellular redox status and inflammatory responses [63,64]. Oxidative stress activates Nrf2, which then migrates into nuclei to bind to antioxidative stress response elements, initiating antioxidant gene transcription and causing HO-1 upregulation [65]. Nrf2 breaks down hemoglobin into products with antioxidant properties. A complex interaction occurs between ROS and the Nrf2/HO-1/NF-κB pathway. ROS can either activate the Nrf2/HO-1 pathway or participate in inflammatory processes by activating the NF-κB pathway. Oxidative stress results in excessive free radical generation to activate NF-κB, which triggers an inflammatory response. Activated Nrf2 and HO-1 inhibit NF-κB to exert anti-inflammatory effects. NF-κB inhibition decreases inflammatory cytokine production, downregulating the inflammatory response. In this study, HO-1 and Nrf2 expression in the M group was downregulated, but LRM treatment upregulated their expression, suggesting that these two factors are related to LRM’s inhibition of alcohol-mediated oxidative stress. The NF-κB pathway was activated after acute alcohol exposure, while the Nrf2/HO-1 pathway was inhibited. LRM had the opposite effect on these two pathways.

## 4. Materials and Methods

### 4.1. Materials and Chemicals

LRM was obtained from Beijing Panda Health Management Co., Ltd. (Beijing, China). Silibinin capsules (No. 250711083) were obtained from Tianjin Tianshili Shengte Pharmaceutical Co., Ltd. (Tianjin, China). Erguotou liquor (containing 56% ethanol) [66,67] (No. 2021120408) was obtained from Beijing Hongxing Limited by Share Ltd. (Beijing, China).

Alanine aminotransferase (ALT), aspartate aminotransferase (AST), and triglyceride (TG) detection kits were obtained from Biosino Bio-Technology and Science Inc. (Beijing, China). Lactate dehydrogenase (LDH), total cholesterol (TC), low-density lipoprotein cholesterol (LDL-C), malondialdehyde (MDA), reactive oxygen species (ROS), superoxide dismutase (SOD), glutathione peroxidase (GST), catalase (CAT), and Interleukin—1β (IL-1β), IL-6, and TNF-α [68,69,70,71,72] detection kits were acquired from Shanghai Enzyme-linked Biotechnology Co., Ltd. (Shanghai, China). Nrf2, HO-1, NF-κB, and TNF-α [48,73] primers, the animal total RNA rapid extraction kit, SynScript^®^III RT SuperMix for qPCR, and ArtiCan^ATM^SYBR qPCR Mix were obtained from Beijing Tsingke Biotech Co., Ltd. (Beijing, China). The remaining reagents and chemicals were analytically pure and locally sourced. Rabbit anti-Nrf2 (BS-1074R), anti-HO-1 (BS-2075R), and anti-NF-κB p65 (BS-0465R) antibodies were acquired from Wuhan Servicebio Technology Co., Ltd. (Wuhan, China).

### 4.2. Animals

C57BL/6J male mice (18–22 g) were obtained from Beijing HFK Bioscience Co., Ltd. (Beijing, China) and raised in a specific pathogen-free (SPF) barrier facility at 20–26 °C and 40–70% humidity with a 12 h light/dark cycle. The animals had free access to food and water. Animal care and research conformed to ethical guidelines and were approved by the animal ethics committee of the Institute of Medicinal Plant Development, Chinese Academy of Medical Sciences (approval number: SLXD-20230602013). Animals were acclimatized for 1 week prior to experimentation.

### 4.3. Grouping and Drug Use

Altogether, 60 male C57BL/6J mice were randomized to the normal control (NC, no-treatment control), model (M, 15 mL physiological saline/kg BW), positive dose (S, 60 mg silibinin/kg BW), and low-, medium-, and high-dose treatment (LL, LM, LH; 50, 125, and 375 mg LRM/kg BW, respectively) groups. LRM, silibinin, or physiological saline were administered consecutively for 21 days. On days 18–21, 1 h after treatment, all mice except those in the NC group were administered 15 mL of alcohol/kg BW. On day 21, the mice were kept without access to water for 6 h after gastric alcohol modeling. The mice were euthanized under isoflurane anesthesia. Blood and liver tissue were collected simultaneously. Serum was obtained through 15 min centrifugation of blood at 4 °C and 2400× *g*. Blood biochemical analyses were performed using 200 µL of serum samples, whereas the remaining samples were preserved at −80 °C. Liver tissue weight was measured, and the liver index was calculated. Afterward, one part of the liver tissue was used for histopathological analyses. The other part was divided into 20 mg pieces and frozen at −80 °C until further analyses. Then, 10% of the tissue homogenate was prepared by adding 10× phosphate-buffered saline (PBS, 200 μL) to 20 mg of liver tissue in an ice water bath with homogenizer model JXFSTPRP-24L (Shanghai Jingxin Industrial Development Co., Ltd., Shanghai, China). After 15 min of centrifugation of the resultant homogenate at 2400× *g* at 4 °C, the supernatants were collected to analyze oxidative stress and inflammation. The remaining liver samples were subjected to gene and protein level analysis.

### 4.4. Total Anthocyanin Content Determination

A modified pH differential approach was adopted to measure the total anthocyanin content (TAC) in the LRM represented by petunia (Pt). Briefly, LRM (0.1 g) was introduced into the extraction solution (50 mL, HCl: 80% EtOH = 3:97, *v*/*v*). Ultrasonic model KQ-500DE (Beijing Boyuan Xiangde Scientific Instrument Co., Ltd., Beijing, China) extraction was performed at 50 °C for 30 min with 3 min of centrifugation at 8000 rpm. Fivefold serial dilutions of the supernatants were obtained to obtain a sample solution. The sample solution was divided into two parts (1 mL), each of which was serially diluted five times with either pH 1.0 or pH 4.5 buffer (0.4 M sodium acetate). The absorbance values at 532 and 700 nm were measured three times. The TAC was represented by the equivalent of petunia (Pt g/100 g) and determined by Formula (1):

(1) $$TAC(Ptg/100g)=\frac{\Delta A\times MW\times DF\times V}{\varepsilon \times l\times m\times 10}$$

where, Δ*A* is the difference between pH 1.0 (A_532_ nm–A_700_ nm) and pH 4.5 (A_532_ nm–A_700_ nm); *MW* represents the molecular weight (Pt’s molar mass is 912.7 g/mol). *DF* indicates the dilution coefficient; *V* represents the volume extracted (mL); ε is the molar extinction coefficient (29,591 for Pt, L·mol^–1^·cm^–1^); 1 is the cupola thickness (1 cm); m is the sample weight in g; and 10 is the conversion factor from g to 100 g [18,74].

### 4.5. Histopathological Analyses of Liver Tissue

After immersion in 4% paraformaldehyde for 12–24 h, the fresh liver tissue was dehydrated with absolute ethyl alcohol, cleared with pure water, and paraffin-embedded before being sliced into 4–5 µm sections for staining with hematoxylin and eosin. Hepatopathologists examined the slides with a 200× microscope supervised by an expert.

### 4.6. Liver Index Analysis

The liver indices were determined as follows:

(2) $$Liver\;index\left(\%\right)=\frac{liver\;mass}{weight}\times 100\%$$

### 4.7. Serum Biochemical Analysis

An AU400 automatic biochemical analyzer (Beckman Coulter Commercial Enterprise (Beijing, China) Co., Ltd.) was used to measure the serum ALT, AST, and TG levels.

ELISA kits were used to measure TC, LDL-C, and LDH levels within the serum following specific protocols.

### 4.8. ROS Analysis

An ELISA kit was used to measure ROS levels in liver tissue following the manufacturer’s protocols.

### 4.9. Liver Oxidative Stress Analysis

SOD, MDA, CAT, and GSH contents in the liver were determined with ELISA kits following specific protocols.

### 4.10. Inflammation Analysis

Liver tissue TNF-α and IL-1β levels were analyzed with ELISA kits following specific protocols.

### 4.11. Gene Levels

Total RNA was extracted from 20 mg of liver specimens via a centrifugal column method (Beijing Tsingke Biotech Co., Ltd.). Using a Nanodrop 2000 ultra microvolume spectrophotometer (Thermo Fisher Scientific, Inc., Waltham, MA, USA), RNA purity was analyzed. An optical density 260/280 ratio of approximately 2.0 indicated high purity.

A SynScript^®^Ⅲ RT SuperMix for qPCR kit was subsequently used to prepare first-strand cDNA. The mixture of RNA and kit reagents was subjected to 15 min of incubation at 50 °C and 5 s at 85 °C to synthesize cDNA.

A quantitative polymerase chain reaction (qPCR) assay was performed using an ArtiCanATM SYBR qPCR kit and an ABI Quantstudio6 Flex Real-Time PCR System (Applied Biosystems Inc., Singapore). qPCR was conducted using specific primers (Table 1) and involved the following steps: 1 min initial denaturation at 95 °C; 10 s denaturation at 95 °C, 20 s annealing at 60 °C, and 1 min final extension at 72 °C for 40 cycles. *GAPDH* served as an endogenous control. Utilizing the cycle threshold (Ct) value, relative gene expression was determined through the 2^−∆∆CT^ methodology.

### 4.12. Immunohistochemistry

To assess the expression of Nrf2, HO-1, and NF-κB proteins, paraffin-embedded liver tissue samples were sectioned into 4µm slices, followed by dewaxing and rehydration. The tissue sections were immersed in 10 µmol/L citrate buffer, microwaved for 15 min, cooled to ambient temperature after repair, and rinsed with PBS three times (5 min each). The water beads were thoroughly mixed and blotted with absorbent paper. Thereafter, a peroxidase blocking solution (50 μL) was added to each section, which was then incubated for 20 min at ambient temperature and rinsed with PBS three times (5 min each). After thoroughly mixing the water beads and blotting with absorbent paper, the tissue sections were blocked using serum prior to 30 min of incubation at ambient temperature. Following serum removal, antibodies against HO-1, Nrf2, NF-κB, and TNF-α, which were diluted at a 1:100 ratio, were added, and the tissue sections were incubated overnight at 4 °C. After washing with PBS three times (5 min each), the sections were incubated for 50 min with horseradish peroxidase-labeled secondary antibodies at ambient temperature. Finally, DAB (Wuhan Servicebio Technology Co., Ltd.) chromogenic solution was added for section staining, and reverse hematoxylin staining was performed to verify the ratio. The sections were then dehydrated and sealed. A Nikon E100 optical microscope (Nikon Co., Tokyo, Japan) was used for slice observation, while ImageJ 1.8.0 (Rawak Software Inc., Stuttgart, Germany) was used for slice analysis.

### 4.13. Statistical Analysis

GraphPad Prism 10.0 was used for one-way analysis of variance. A least significant difference approach was used to perform multiple comparisons of the two groups. The experimental data are presented as the mean ± standard deviations. *p* < 0.05 indicated a significant statistical difference. Plots were drawn using GraphPad Prism 10.0.

## 5. Conclusions

The study demonstrated that LRM significantly improved liver histopathology and liver index while also reducing AST and ALT liver function indicators. LRM effectively reduced damage caused by reactive oxygen species by lowering ROS and MDA levels, addressing issues such as steatosis. Furthermore, LRM enhanced antioxidant capacity, decreased inflammatory response factors, regulated Nrf2 and HO-1 at the transcriptional level, and downregulated NF-κB and TNF-a gene and proteins expression. LRM effectively prevents and treats ALD through the Nrf2/HO-1/NF-KB pathway. These results suggest that LRM’s multifaceted protective mechanisms hold promise for ALD prevention and treatment.

This study provides a preliminary exploration of LRM’s impact on ALD. It is worth noting that this mechanism is just one of the potential pathways considering liver tissue’s intricate and varied functions. Future research could delve deeper into the endotoxin-induced damage and apoptosis inhibition resulting from excessive alcohol intake, as well as thoroughly investigate the LRM mechanism.

## Figures and Tables

**Table 1:** Reverse transcription‒qPCR primer sequences.

Gene	Species	Forward (5′–3′)	Reverse (3′–5′)
*Nf-κb p65*	Mouse	CTTCTGGGCCTTATGTGGAGATC	GGTCCTGTGTAGCCATTGATCTT
*Ho-1*	Mouse	GCTCGAATGAACACTCTGGAGAT	ACTCTGGTCTTTGTGTTCCTCTG
*Nrf2*	Mouse	CAGAGTGATGGTTGCCCACT	CACACACTTTCTGCGTGCTC
*Tnf-α*	Mouse	GACCCCTCACACTCAGATCATCTT	CCTTGAAGAGAACCTGGGAGTAG
*Il-1β*	Mouse	TCCACCTCAATGGACAGAATATC	CCGTCTTTCATTACACAGGACA
*Gapdh*	Mouse	ACTCTTCCACCTTCGATGCC	TGGGATAGGGCCTCTCTTGC

## Data Availability

Data are included in the current research.
